# Adverse obstetrical outcomes for women with endometriosis and adenomyosis: A large cohort of the Japan Environment and Children’s Study

**DOI:** 10.1371/journal.pone.0220256

**Published:** 2019-08-02

**Authors:** Takashi Harada, Fuminori Taniguchi, Hiroki Amano, Youichi Kurozawa, Yuki Ideno, Kunihiko Hayashi, Tasuku Harada, Japan Environment and Children’s Study Group

**Affiliations:** 1 Department of Obstetrics and Gynecology, Tottori University Faculty of Medicine, Yonago, Japan; 2 Department of Public Health, Tottori University Faculty of Medicine, Yonago, Japan; 3 Department of Laboratory Science and Environmental Health Sciences, Graduate School of Health Sciences, Gunma University, Maebashi, Japan; Ospedale dei Bambini Vittore Buzzi, ITALY

## Abstract

**Background:**

Because of the increased number of diagnosed cases of endometriosis or adenomyosis resulting in infertility, many women require assisted reproductive technology (ART) to become pregnant. However, incidences of obstetric complications are increased for women who conceive using ART. There has been no prospective cohort study examining the influence of endometriosis and adenomyosis on obstetric outcomes after adjusting for the confounding influence of ART therapy.

**Objective:**

This study evaluated the impact of endometriosis and adenomyosis on the incidence of adverse pregnancy outcomes.

**Study design:**

Data were obtained from a prospective cohort study, known as the Japan Environment and Children’s Study (JECS), of the incidence of obstetric complications for women with endometriosis and adenomyosis. The data of 103,099 pregnancies that resulted in live birth or stillbirth or that were terminated through abortion between February 2011 and July 2014 in Japan were included.

**Results:**

Women with endometriosis or adenomyosis were at increased risk for complications during pregnancy compared to those without a medical history of endometriosis (odds ratio [OR], 1.32; 95% confidence interval [CI], 1.23 to 1.41) or adenomyosis (OR, 1.72; 95% CI, 1.37 to 2.16). Our analysis showed that the adjusted ORs for obstetric complications of pregnant women who conceived naturally or after infertility treatment that did not involve ART therapy were 1.26 (CI, 1.17 to 1.35) for pregnant women with a history of endometriosis and 1.52 (CI, 1.19 to 1.94) for those with a history of adenomyosis.

**Conclusions:**

The presence of endometriosis and adenomyosis significantly increased the prevalence of obstetric complications after adjusting for the influence of ART outcomes.

## Introduction

Endometriosis is a chronic inflammatory disease characterized by the presence of extrauterine endometrial-like tissue. Prevalence of endometriosis has increased up to 50% in women with infertility [1]. Various pathogenetic mechanisms of infertility due to the presence of endometriosis have been indicated [2]. As a possible etiology, the abnormal eutopic endometrium of women with endometriosis may play an important role by exhibiting subtle but biologically important molecular abnormalities, such as an enhanced production of estrogen, cytokines, prostaglandins, and metalloproteinases [3,4].

Adenomyosis is a benign uterine disorder, characterized by the presence of endometrial glands and stroma deep within the myometrium. Adenomyosis has peak prevalence during reproductive ages [5]. Until recently, it was considered that adenomyosis is associated with multiparity, but not impaired implantation during *in vitro* fertilization (IVF) treatment [6]. In contrast, Dueholm demonstrated that the presence of adenomyosis is associated with a significant reduction in implantation of ‘good quality’ embryos in women undergoing IVF treatment [7].

It appears that women with endometriosis or adenomyosis are more likely to struggle with achieving pregnancy and to undergo infertility treatments, including assisted reproductive technology [8]. In addition, it is well established that singleton pregnancies conceived by ART are at a higher risk of complications than those conceived naturally [9]. In the present study, we assessed the pregnancy outcomes of women with or without gynecological disorders after excluding the age adjusted influence of ART therapy.

## Materials and methods

### Data sources

The Japan Environment and Children’s Study (JECS) is a national project, designed to improve children’s health and development. A total of 100,000 children and their parents across 15 regions in Japan have participated in it [10]. The purpose of the JECS, an ongoing prospective birth cohort study that began in 2011, is to evaluate the impact of various environmental factors on children’s health and development [11,12]. The JECS protocol was approved by the Institutional Review Board (IRB) on epidemiological studies of the Ministry of the Environment (MOE) and the Ethics Committees of all participating institutions. The present study was based on a dataset released in June 2016 that did not contain patient-identifying information. Enrollment of participants was conducted between January 2011 and March 2014. As stated above, the jecs-ag-20160424 dataset does not contain any patient identifying information. All participants provided their written informed consent.

In this study, each woman completed a questionnaire regarding her history of gynecological disorders, recording whether she had been diagnosed during the past year and/or had undergone infertility treatment. The gynecological diseases described in the questionnaire included endometriosis, adenomyosis, uterine myoma, ovarian tumor, and congenital uterine anomaly. This study did not consider the time period between diagnosis of the gynecological disorder and pregnancy. Further data concerning obstetrical complications and neonatal outcomes were collected from medical records at the institutions that provided obstetric care to these patients.

### Participants

Women who gave birth, experienced stillbirth, or whose pregnancy was terminated through abortion were included in the JECS, with participants enrolled before delivery (or termination). A total of 103,099 pregnancies were reported. The exclusion criteria included multiple pregnancies, as well as pregnant women who could not clearly articulate their gynecological history. This study contained a total of 96,655 women. The presence of endometriosis or adenomyosis was based on the responses to a self-reported questionnaire.

### Outcomes and covariates

Women’s age was recorded at the time of delivery or pregnancy outcome and categorized as <20, 20–24, 25–29, 30–34, 35–39, or ≥40 years. The women were also classified as smokers, ex-smokers, and non-smokers. Their smoking habits were classified as <3 days/week and ≥3 days/week. Based on alcohol consumption, women were classified as non-drinkers, ex-drinkers, and current drinkers. ART therapy included IVF, intracytoplasmic sperm injection (ICSI), frozen-thawed embryo transfer, and blastocyst embryo transfer. ART did not include intra-uterine insemination.

Complications of pregnancy were characterized as spontaneous abortion, extremely preterm birth (22–27 weeks gestation), preterm birth (28–36 weeks gestation), premature rupture of the membranes (PROM), gestational diabetes, preeclampsia, placenta previa, placental abruption, fetal growth restriction (FGR), and non-reassuring fetal status (NRFS). Perinatal mortality was defined as live-birth, abortion, and stillbirth.

### Diagnostic criteria for obstetrical complications

The medical definitions and diagnostic criteria of obstetrical complications have been described previously [13, 14].

### Statistical analysis

The Wilcoxon rank-sum test or the chi-squared test was used to evaluate significant differences in age, smoking status, passive smoking, alcohol consumption, gestational age, and other clinical characteristics between women who had been diagnosed with a gynecological disorder and those with no such diagnosis. A chi-squared test, Fisher’s exact test, or logistic regression analysis was used to compare the incidences of pregnancy complications between the two groups. To examine the associations between gynecological disorders and fertility treatment, all women were classified into the following two groups: group A1 (the reference group), which included women with no history of gynecological disorders and group A2, which included women with gynecological disorders who had not undergone infertility treatment (Table 1). Unconditional logistic regression models were used to estimate age-adjusted odds ratios (ORs) and their 95% confidence intervals (CIs). To examine the interactions between gynecological disorders and ART therapy, women were also grouped into the following two groups: group B1 (the reference group), which included women without a history of gynecological disorders who had conceived naturally or through infertility treatment but without ART therapy, and group B2, which included women with gynecological disorders who had not undergone ART therapy. These analyses were restricted to pregnancies with complete covariate data. All analyses were performed using SAS V.9.4 (SAS Institute Inc., Cary, NC USA.) A *P* value of <0.05 was considered significant for all statistical analyses.

**Table 1:** Summary of groups to analyze the interaction between gynecological disorders and fertility treatment.

Group	Women’s medical background			
	History of gynecological disorder	Natural conception	infertility treatment except for ART	ART therapy
**A1**	**-**	**+**	**-**	**-**
**A2**	**+**	**+**	**-**	**-**
**B1**	**-**	**+**	**+**	**-**
**B2**	**+**	**+**	**+**	**-**

## Results

A total of 96,655 pregnant women were enrolled between January 2011 and March 2014 (Table 2).

**Table 2:** Obstetrical characteristics of women with and without gynecological disorders.

	Endometriosis					Adenomyosis				
	Positive (n = 3,517)		Negative (n = 93,138)		P Value	Positive (n = 325)		Negative (n = 96,330)		P Value
	n	%	n	%		n	%	n	%	
**Gestational age**										
weeks, median [range]	39 [10–42]		39 [6–43]		<0.001^b^	38 [16–41]		39 [6–43]		<0.001^b^
< 22 W	20	0.6	563	0.6		6	1.9	577	0.6	
Spontaneous abortion	13		390			4		399		
Induced abortion	6		136			2		140		
Unknown	1		37			0		38		
22–27 W	21	0.6	269	0.3		4	1.2	286	0.3	
28–36 W	214	6.1	4,175	4.5		45	13.9	4,344	4.5	
37–41 W	3,257	92.6	87,903	94.4		270	83.0	90,890	94.4	
≥ 42 W	5	0.1	214	0.2		0	0	219	0.2	
Missing data	0		14			0		14		
**Mode of delivery**										
Vaginal delivery	2,586	73.9	75,354	81.5	<0.001^a^	206	64.0	77,734	81.2	<0.001^a^
Cesarean section	915	26.1	17,151	18.5		116	36.0	17,950	18.8	
Missing data	16		633			3		646		
**Infertility treatment**										
No	2,705	77.1	85,345	92.0	<0.001^a^	209	64.7	87,841	91.6	<0.001^a^
Yes	802	22.9	7,368	8.0		114	35.3	8,056	8.4	
Ovulation induction	442		4,517			62		4,897		
Artificial insemination	249		2,042			27		2,264		
ART	411		2,616			59		2,968		
ICSI	198		1,222			29		1,391		
Blastocyst transfer	131		731			15		847		
Other	131		1,185			16		1,300		
Missing data	10		425			2		433		

Data expressed as n (%)

^a^, Chi-squared test

^b^, Wilcoxon rank-sum test

The number of women diagnosed with endometriosis and adenomyosis were 3,517 and 325, respectively. There were 3,381 women with a history of endometriosis, 189 of adenomyosis and 136 with that of both disorders. The frequency of spontaneous abortions in women with adenomyosis was greater than that in pregnant women without adenomyosis (1.9% vs. 0.6%). The rate of preterm delivery between 22 and 36 weeks of gestational age in women with endometriosis or adenomyosis was higher than that in women without these diseases (6.7% vs. 4.8%, and 15.1% vs. 4.8%, respectively). The rate of cesarean delivery was higher in women who were diagnosed with either disease. Of the 3,517 pregnant women with a reported diagnosis of endometriosis before pregnancy, 2,705 conceived naturally (77.1%) and 411 conceived following ART therapy (11.7%). On the other hand, of the 325 women with a reported diagnosis of adenomyosis before pregnancy, 209 conceived naturally (64.7%) and 59 received ART therapy (18.2%).

Table 3 shows the number of obstetrical complications in patients with endometriosis or adenomyosis. The frequency of obstetric complications was 53.6% (1,884/3,517) in women with endometriosis and 60.0% (195/325) in women with adenomyosis. The incidence rates of preterm PROM, gestational diabetes, and placenta previa were higher in women diagnosed with endometriosis or adenomyosis. Only pregnant women with a medical history of adenomyosis experienced adverse events of mild preeclampsia, placental abruption, FGR, and fetal death.

**Table 3:** Types of obstetrical complications and neonatal outcomes.

		Endometriosis					Adenomyosis				
		Positive		Negative		P Value	Positive		Negative		P Value
		n	%	N	%		n	%	n	%	
**Obstetrical complications**											
Negative		1,633	46.4	50,308	54.0	<0.001^a^	130	40.0	51,811	53.8	<0.001^a^
Positive		1,884	53.6	42,830	46.0		195	60.0	44,519	46.2	
**Premature rupture of membranes**											
Negative		3,197	90.9	85,415	91.7	0.093^a^	287	88.3	88,325	91.7	0.034^a^
Positive	Preterm PROM	68	1.9	1,102	1.2		15	4.6	1,155	1.2	
	Term PROM	223	6.3	5,804	6.2		19	5.9	6,008	6.2	
	Unknown	29	0.8	817	0.9		4	1.2	842	0.9	
**Gestational diabetes**											
Negative		3,394	96.5	90,667	97.3	0.003^a^	309	95.1	93,752	97.3	0.023^a^
Positive		123	3.5	2,471	2.7		16	4.9	2,578	2.7	
**Preeclampsia (mild)**											
Negative		3,426	97.4	91,039	97.7	0.204^a^	310	95.4	94,155	97.7	0.013^a^
Positive		91	2.6	2,099	2.3		15	4.6	2,175	2.3	
**Preeclampsia (severe)**											
Negative		3,475	98.8	92,257	99.0	0.133^a^	322	99.1	95,410	99.0	1.000^a^
Positive		42	1.2	881	1.0		3	0.9	920	1.0	
**Placenta previa**											
Negative		3,454	98.2	92,619	99.5	<0.001^a^	320	98.5	95,753	99.4	0.048^a^
Positive		63	1.8	519	0.5		5	1.5	577	0.6	
**Abruption of the placenta**											
Negative		3,498	99.5	92,728	99.6	0.364^a^	321	98.8	95,905	99.6	0.058^a^
Positive		19	0.5	410	0.4		4	1.2	425	0.4	
**Fetal growth restriction**											
Negative		3,431	97.5	91,260	98.0	0.078^a^	307	94.5	94,384	98.0	<0.001^a^
Positive		86	2.5	1,878	2.0		18	5.5	1,946	2.0	
**Non-reassuring fetal status**											
Negative		3,433	97.6	90,854	97.5	0.868^a^	312	96.0	93,975	97.6	0.101^a^
Positive		84	2.4	2,284	2.5		13	4.0	2,355	2.4	
**Perinatal mortality**											
Livebirth		3,486	99.1	92,335	99.1	0.779^a^	315	96.9	95,506	99.2	<0.001^a^
Stillbirth / Abortion		31	0.9	791	0.9		10	3.1	812	0.8	
missing		0		12			0		12		

Data expressed as n (%)

^a^, Fisher’s exact test

In multivariable analysis, maternal age, smoking habits, passive smoking and alcohol consumption were included as potential risk factors for adverse pregnancy outcomes. As shown in Table 4, women with endometriosis were at a higher risk of obstetrical complications relative to those without endometriosis, following adjustment for the confounding characteristics (adjusted odds ratio: aOR = 1.32; 95% confidence interval: CI = 1.23–1.41). Particularly, the rates of extremely preterm birth, preterm birth, preterm PROM, and placenta previa were higher in women with endometriosis (aOR = 1.97, aOR = 1.32, aOR = 1.62, and aOR = 2.87, respectively). The aOR for GDM was 1.11 (CI = 0.92–1.35).

**Table 4:** Odds ratios of obstetrical complications in women with endometriosis.

	Endometriosis							
	Crude OR	95% CI			aOR	95% CI		
Obstetrical complications	1.36	1.27	-	1.45	1.32	1.23	-	1.41
Extremely preterm birth	2.08	1.33	-	3.24	1.97	1.26	-	3.09
Preterm birth (28–36 W)	1.38	1.20	-	1.59	1.32	1.15	-	1.53
Preterm PROM	1.65	1.29	-	2.11	1.62	1.27	-	2.08
Placenta previa	3.26	2.50	-	4.24	2.87	2.19	-	3.75

Note: aOR, adjusted odds ratio; CI, confidence interval

Multivariable-adjusted by age, smoking, passive smoking, alcohol drinking

On the other hand, women with adenomyosis had increased risk of obstetrical complications compared to those without adenomyosis (aOR = 1.72; 95% CI = 1.37–2.16) (Table 5). The odds of extremely preterm birth, preterm birth, and preterm PROM appeared to increase in women with either endometriosis or adenomyosis. Interestingly, pregnant women with adenomyosis, but not endometriosis, had a high risk of preeclampsia (mild), placental abruption and FGR compared to those without adenomyosis (aOR = 1.86, aOR = 2.62, and aOR = 2.72, respectively). The GDM and placenta previa rates were not higher for women with adenomyosis after adjustment for the confounding characteristics. The OR for spontaneous abortion was 2.51 (CI = 0.93–6.79).

**Table 5:** Odds ratios of obstetrical complications in women with adenomyosis.

	Adenomyosis							
	Crude OR	95% CI			aOR	95% CI		
Obstetrical complications	1.74	1.39	-	2.17	1.72	1.37	-	2.16
Extremely preterm birth	4.18	1.55	-	11.29	3.63	1.34	-	9.83
Preterm birth (28–36 W)	3.40	2.48	-	4.67	2.95	2.14	-	4.09
Preterm PROM	3.99	2.37	-	6.72	3.74	2.22	-	6.32
Preeclampsia (mild)	2.10	1.25	-	3.53	1.86	1.11	-	3.14
Abruption of the placenta	2.81	1.05	-	7.57	2.62	0.97	-	7.07
Fetal growth restriction	2.84	1.76	-	4.58	2.72	1.67	-	4.46

Note: aOR, adjusted odds ratio; CI, confidence interval

Multivariable-adjusted by age, smoking, passive smoking, alcohol drinking

To separate the influences of gynecological disorders from the effects of infertility treatment on the analysis, two combined groups were evaluated using a logistic regression analysis. A summary of the groups for analyzing the interactions between gynecological disorders and fertility treatment is shown in Table 1. Among the pregnant women who conceived naturally, the aORs of extremely preterm birth, preterm birth, preterm PROM, and placenta previa in women diagnosed with endometriosis (Group-A2) were higher than those in women without endometriosis (Group-A1) (Table 6). In women with endometriosis who conceived naturally or after infertility treatment without ART therapy (Group-B2), the aOR for obstetrical complications was 1.26 (95% CI = 1.17–1.35), and the aORs for extremely preterm birth, preterm birth, preterm PROM, and placenta previa associated with endometriosis were 2.15 (95% CI = 1.35–3.44), 1.28 (95% CI = 1.10–1.49), 1.52 (95% CI = 1.16–2.00), and 2.11 (95% CI = 1.51–2.94), respectively.

**Table 6:** Odds ratios of having endometriosis associated with infertility treatment.

**(A)**							
		A1	A2				
	**Past history of endometriosis**	Negative	Positive				
	**Fertility treatment**	Negative	Negative				
			aOR	95% CI			
	Obstetrical complications	ref	1.25	1.16	-		1.35
	Extremely preterm birth	ref	2.13	1.28	-		3.54
	Preterm birth (28–36 W)	ref	1.29	1.10	-		1.53
	Preterm PROM	ref	1.57	1.18	-		2.10
	Placenta previa	ref	2.32	1.65	-		3.27
**(B)**							
		B1	B2				
	**Past history of endometriosis**	Negative	Positive				
	**Fertility treatment**	ART negative	ART negative				
			aOR	95% CI			
	Obstetrical complications	ref	1.26	1.17	-	1.35	
	Extremely preterm birth	ref	2.15	1.35	-	3.44	
	Preterm birth (28–36 W)	ref	1.28	1.10	-	1.49	
	Preterm PROM	ref	1.52	1.16	-	2.00	
	Placenta previa	ref	2.11	1.51	-	2.94	

Estimates are based on models adjusted for age. Note: aOR, adjusted odds ratio

CI, confidence interval; n/a, not applicable

In terms of adenomyosis, the aOR for obstetrical complications in pregnant women with adenomyosis who conceived naturally or after infertility treatment without ART therapy (Group-B2) was 1.52 (95% CI = 1.19–1.94) (Table 7). In addition, our data showed that group B2 had higher frequencies of extremely preterm birth, preterm birth, preterm PROM, placental abruption, and FGR: odds ratio = 4.76 (95% CI = 1.75–12.91), 2.57 (95% CI = 1.77–3.75), 2.80 (95% CI = 1.43–5.46), 3.29 (95% CI = 1.22–8.89), and 2.88 (95% CI = 1.70–4.86), respectively. The aOR for mild preeclampsia was not higher for women with adenomyosis who conceived naturally or underwent infertility treatment without ART therapy (group B2).

**Table 7:** Odds ratios of having adenomyosis associated with infertility treatment.

**(A)**						
		A1	A2			
	**Past history of adenomyosis**	Negative	Positive			
	**Fertility treatment**	Negative	Negative			
			OR	95% CI		
	Obstetrical complications	ref	1.28	0.98	-	1.69
	Extremely preterm birth	ref	6.16	2.26	-	16.79
	Preterm birth (28–36 W)	ref	2.30	1.48	-	3.59
	Preterm PROM	ref	2.77	1.30	-	5.91
	Abruption of the placenta	ref	3.11	0.99	-	9.76
	Fetal growth restriction	ref	2.45	1.30	-	4.64
**(B)**						
		B1	B2			
	**Past history of adenomyosis**	Negative	positive			
	**Fertility treatment**	ART negative	ART negative			
			OR	95% CI		
	Obstetrical complications	ref	1.52	1.19	-	1.94
	Extremely preterm birth	ref	4.76	1.75	-	12.91
	Preterm birth (28–36 W)	ref	2.57	1.77	-	3.75
	Preterm PROM	ref	2.80	1.43	-	5.46
	Abruption of the placenta	ref	3.29	1.22	-	8.89
	Fetal growth restriction	ref	2.88	1.70	-	4.86

Estimates are based on models adjusted for age. Note: aOR, adjusted odds ratio

CI, confidence interval; n/a, not applicable

## Discussion

Our results demonstrated two important clinical observations. First, the pregnant women with a past history of endometriosis and adenomyosis, regardless of whether being conceived after ART therapy, have an increased risk of obstetrical complications. Second, the types of obstetrical complications in women diagnosed with adenomyosis are different from the pregnancy outcomes of women with endometriosis.

This is the first study that reports obstetric complications in women with adenomyosis, after excluding the influence of ART therapy. Women with adenomyosis are more likely to struggle with achieving pregnancy and to receive infertility treatment, including ART therapy. It was previously demonstrated that women with adenomyosis who conceived using ART therapy were at high risk of perinatal and maternal complications, such as preterm delivery, preeclampsia, placenta previa and placenta abruption [15,16]. In addition, maternal factors associated with infertility may contribute to adverse outcomes rather than the ART procedures themselves [9].

We presented that placenta previa was more frequent in women with a history of endometriosis after adjusting for the influence of age and ART therapy. Our results support previous evidence which demonstrated that pregnant women with endometriosis had an increased risk of placenta previa [14, 17–19]. This increased risk of placenta previa suggested that progesterone resistance and inadequate uterine contractibility in endometriosis may be involved in deferred implantation and embryo displacement [19].

We have previously shown that preterm PROM and premature delivery were also frequent in women with a history of endometriosis [14]. The etiological causes of preterm delivery due to pre-existing endometriosis may be explained by different mechanisms: endometriosis-related chronic inflammation that makes tissues and vessels more friable [20], inadequate uterine contractility [21] and the alterations in uterine junctional zone (JZ) [22] in women with endometriosis. The current study showed that women with a history of adenomyosis are identical to those with endometriosis with respect to their high risk of preterm delivery and preterm PROM after adjusting for the confounding influence of ART therapy. The risk of preterm birth and preterm PROM in pregnant women with adenomyosis were 2-fold higher than those in women with endometriosis. The effect of concomitant adenomyosis on preterm delivery has been previously evaluated in only two studies which examined the relationship between adenomyosis and preterm birth, and demonstrated an increased risk of preterm birth in adenomyosis [23,24]. The mechanism of preterm PROM in women with adenomyosis can be explained by the failure of physiologic transformation of spiral arteries in the inner myometrial segment, termed as JZ. There was no difference in myometrial spiral artery remodeling according to the presence or absence of histological chorioamnionitis among patients with preterm labor [25]. Noninfectious etiologies, such as placental hypoperfusion also appeared to increase production of proinflammatory mediators and were the leading cause of preterm delivery [26].

We also elucidated that, unlike the risks of obstetrical complications in women with endometriosis, the risks of FGR and placental abruption were also significantly higher in women with adenomyosis. It was previously shown that blood flow within the adenomyosis lesions is abundant, while the placenta has diminished blood flow based on the results of blood flow measurements in the myometrium and placenta of women with adenomyosis and severe FGR [27]. One hypothesis is that the placental hypoperfusion that results in a small placenta may lead to presence of FGR. Several researchers have reported that a substantially increased risk of abruption occurs when placental membranes rupture pre-term [28,29]. In this study, the risk of preterm birth in the pregnant women diagnosed with adenomyosis was much higher than that in women with endometriosis. It is likely that the elevated risk of placental abruption in pregnant women with adenomyosis is due to the high incidence of preterm PROM.

The absence of physiologic transformation of the spiral artery, introduced as defective deep placentation, was the common pathogenesis in these obstetrical complications. Alterations of the JZ in women with endometriosis and adenomyosis can influence vascular resistance of JZ spiral arteries to the onset of decidualization and lead to an increased risk of insufficiently deep placentation [30]. The restriction of physiologic transformation of the spiral artery was believed to be important in miscarriage and, possibly, lower degree of hyperoxia may be a predisposition to later fetal death [31,32]. The absence of physiological transformation of blood vessels by defective deep placentation results in FGR [33]. A restriction in myometrial spiral artery remodeling can contribute to placental abruption by increasing the velocity of blood flow from the uterine artery [34].

Defective remodeling of the myometrial segment was first described in patients with pre-eclampsia, alone or in combination with FGR [35]. Brosens reported that >90% of spiral arteries in the JZ changed physiologically during normal pregnancy compared with 10% in pregnant women with severe preeclampsia [36]. The reason why some patients with defective deep placentation have preeclampsia whereas others have preterm labors was that the extent of vascular pathology is distributed far more widespread in preeclampsia than in preterm birth [25]. In this study, the adjusted OR of mild preeclampsia in women with adenomyosis regardless of ART therapy was 1.76. It is likely that women with preterm births may have developed preeclampsia later if they remained pregnant to term.

Endometriosis and adenomyosis are characterized by the presence of ectopic endometrium, but are also associated with functional and structural changes in the eutopic endometrium and inner myometrium. Both transvaginal ultrasound and magnetic resonance imaging (MRI), especially T2-weighted images, are increasingly used for diagnostic imaging for adenomyosis. The image findings for adenomyosis include asymmetric thickening of the myometrium, myometrial cysts, linear striations radiating out from the endometrium, loss of a clear endomyometrial border, and increased myometrial heterogeneity [37]. It is generally considered that JZ thickening to more than 12 mm is a diagnostic criterion for adenomyosis [38]. The JZ of women with endometriosis was thicker than that of women without endometriosis. There was a positive correlation between the posterior JZ thickness and the stage of endometriosis. Women with the 4 stages of endometriosis were more likely to have a thicker JZ than those with other stages of endometriosis (American Fertility Society, AFS stages 1, 2 and 3) [39]. Due to the fact that the JZ thicknesses were different among women with endometriosis, depending on the stages, and among women with adenomyosis, the types of obstetrical complications observed in pregnant women with endometriosis and those women with adenomyosis were different.

One of the limitations of this study is that whether the diagnoses of endometriosis or adenomyosis were definitive based on the findings of surgery is unknown. There was little information on endometriosis and adenomyosis in the patient’s medical records, and we did not utilize the past medical records of participating women. In addition, this study did not take account for the timing when women with endometriosis and adenomyosis were treated prior to pregnancy. Therefore, it was unclear as to how many of the 96,655 women had apparent findings of pelvic endometriosis or deformed uterine cavity induced by adenomyosis before implantation.

## Conclusions

The present study demonstrated that obstetrical complications such as preterm birth and preterm PROM were more frequent in women with a medical history of endometriosis or adenomyosis. Women who had been diagnosed with endometriosis also had a high incidence of placenta previa. Adenomyosis affected spontaneous abortion, placental abruption and FGR. This study is the first report on obstetrical complications based on the analysis of common factors that show an impact of endometriosis and adenomyosis after adjusting for the confounding influence of ART.

## Supporting information

S1 Table Clinical characteristics of women with and without gynecological disorders. (PDF)
