# Supplementary material for: Adverse obstetrical outcomes for women with endometriosis and adenomyosis: A large cohort of the Japan Environment and Children’s Study
Source: PLoS One. 2019 Aug 2;14(8):e0220256. doi: 10.1371/journal.pone.0220256 (PMC6677302; doi:10.1371/journal.pone.0220256)
Supplement: S1 Table — (PDF) [file pone.0220256.s001.pdf]

S1 Table. Clinical Characteristics of Women with and without Gynecological Disorders

	Endometriosis					Adenomyosis				
	Positive (n=3,517)		Negative (n=93,138)		P Value	Positive (n=325)		Negative (n=96,330)		P Value
	n	%	n	%		n	%	n	%	
**Maternal age (y)**										
< 20	7	0.2	1,084	1.2	<0.001[b]	0	0	1,091	1.2	<0.001[b]
20-24	150	4.3	9,600	10.5		5	1.6	9,745	10.3	
25-29	783	22.7	27,157	29.7		44	13.8	27,896	29.5	
30-34	1,318	38.1	31,767	34.8		101	31.6	32,984	34.9	
35-39	1,004	29.1	18,554	20.3		131	40.8	19,427	20.6	
≥ 40	193	5.6	3,148	3.5		39	12.2	3,302	3.5	
Missing	62		1,828			5		1,885		
**Parity**										
Primipara	1,529	44.5	36,439	40.0	<0.001[a]	152	48.4	37,816	40.1	0.003[a]
Multipara	1,910	55.5	54,656	60.0		162	51.6	56,404	59.9	
Missing	78		2,043			11		2,110		
**Smoking**										
Non-	1,945	55.6	53,918	58.3	0.006[a]	187	57.7	55,676	58.2	0.725[a]
Ex-	1,373	39.3	34,045	36.8		124	38.3	35,294	36.9	
Current	178	5.1	4,471	4.8		13	4.0	4,636	4.9	
Missing	21		704			1		724		
**Passive smoking**										
≤ 3 days/week	2,636	75.1	67,918	73.3	0.018[a]	255	79.2	70,299	73.3	0.018[a]
> 3 days/week	874	24.9	24,748	26.7		67	20.8	25,555	26.7	
Missing	7		472			3		476		
**Alcohol drinking**										
Non-	1,153	32.9	32,187	34.7	0.036[a]	118	36.4	33,222	34.7	0.699[a]
Ex-	2,016	57.5	51,302	55.4		172	53.1	53,146	55.4	
Current	335	9.6	9,214	9.9		34	10.5	9,515	9.9	
Missing	13		435			1		447		

Data expressed as n (%)
a, Chi-squared test
b, Wilcoxon rank-sum test
